# Establishing a Standardized Measure of Quality in Pediatric Liver Surgery: Definition and Validation of Textbook Outcome With Associated Predictors

**DOI:** 10.3389/fsurg.2021.708351

**Published:** 2021-07-21

**Authors:** Juri Fuchs, Katrin Hoffmann, Anastasia Murtha-Lemekhova, Markus Kessler, Patrick Günther, Giovanni Frongia, Pascal Probst, Arianeb Mehrabi

**Affiliations:** ^1^Department of General, Visceral and Transplantation Surgery, University of Heidelberg, Heidelberg, Germany; ^2^Division of Pediatric Surgery, Department of General, Visceral and Transplantation Surgery, University of Heidelberg, Heidelberg, Germany; ^3^Department of General, Visceral, Thorax, Pediatric and Endocrine Surgery, Johannes Wesling Hospital Minden, Heidelberg, Germany

**Keywords:** pediatric liver resection, evidence-based medicine, surgical outcomes, composite outcome measures, pediatric hepatobiliary surgery

## Abstract

**Purpose:** To establish comparable reporting of surgical results in pediatric liver surgery, the recently introduced composite outcome measures Textbook Outcome (TO) and Comprehensive Complication Index (CCI) are applied and validated in a pediatric surgery context for the first time. In a representative cohort of pediatric patients undergoing liver resection, predictive factors for TO and CCI are investigated, and outcomes are compared to available literature on surgical outcomes of pediatric liver resection.

**Methods:** All liver resections for patients under 21 years of age performed at the Department of General, Visceral, Transplantation and Pediatric Surgery of the University of Heidelberg between 2009 and 2020 were included in the analysis. Criteria for TO were defined prior to the analysis. Univariate and Multivariate regression was applied to identify factors associated with TO and CCI.

**Results:** Fifty-three pediatric patients underwent liver resections during the observation period. No 30- or 90-day mortality occurred. Twenty-three patients (43.4%) had a TO. CCI and TO showed highly significant correlation (*b* = −30.33, 95% CI [−37.44; −23.22], *p* < 0.001). Multivariate analyses revealed significant association between intraoperative blood loss (adjusted for circulating blood volume) and CCI (*b* = 0.70, 95%CI [0.22; 1.32], *p* = 0.008) and failure to achieve TO (OR = 0.85, 95%CI [0.69; 0.97], *p* = 0.048).

**Conclusion:** TO and CCI are suited outcome measures in pediatric surgical studies and offer objective comparability of results. Their application in clinical studies will be a major step forward to establish evidence-based therapies in pediatric surgery. Systematic utilization of TO and CCI can aid in generating comparable studies on surgical techniques and outcomes in pediatric liver resection.

## Introduction

Indications for liver resection in children and adolescents are infrequent and, therefore, critical. Liver tumors are often diagnosed late as symptoms are mostly unspecific or entirely absent (1, 2). A multidisciplinary approach is essential, in which the quality of the surgical procedure is the key factor for favorable clinical outcomes (1–4).

Two thirds of liver tumors in pediatric patients are malignant (2, 5). Hepatoblastoma and (fibrolamellar) hepatocellular carcinoma are the most frequent entities (5, 6). Tumors such as biliary rhabdomyosarcoma, angiosarcoma, undifferentiated sarcoma, and germ cell tumors as well as surgery for liver metastases of non-hepatic cancers are rare (5–7). Benign liver tumors in pediatric patients may lead to compression of neighboring organs or vessels, compromise liver function or may be of uncertain biological behavior, and thus necessitate liver resection (5, 7–9).

Evidence of surgical treatment in pediatric patients with surgical liver disease is often based on small case series with low patient numbers. Investigations on surgical aspects in large cohorts are primarily reported by East Asian centers (10, 11). Only few publications on liver resection and surgery specific outcomes in pediatric patients from Western countries exist, with reported study populations ranging from 9 to 128 patients (3, 12–14). Complication rates vary considerably, with surgery-related mortality ranging from 0 to 6% and morbidity rates from 14 to 69% (2, 3, 10, 14–19). Most of these studies apply different, often incomplete outcome measures and varying definitions of surgical quality, ensuing poor comparability, and lowering the quality of conclusions from these results. As children is an especially vulnerable population, comparability of studies originating from different areas must be improved to achieve high quality evidence.

In this context, we have chosen Textbook Outcome (TO) and the Comprehensive Complication Index (CCI) as primary study endpoints and standardized outcome measures, both novel parameters in the pediatric population. We present the first-ever definition and application of TO in pediatric surgery. These outcome measures have recently been introduced in clinical research on adult surgical patients. They have now been frequently used and proven to be more valid and robust parameters of surgical quality compared to single outcomes variables in many different populations (20). The advantage of these composite endpoints is the comprehensive assessment of the quality of a medical procedure, instead of cherry-picking single components of patient's health condition (20–22).

We present one of the largest single-center cohorts of pediatric patients undergoing liver resection hitherto reported, with representative patient characteristics and comprehensive peri- and postoperative data. The aim is to introduce the newly defined Textbook Outcome as a comparable outcomes measure in pediatric liver surgery.

## Methods

### Ethical Approval and Data Acquisition

The institutional review board of the Medical Faculty of the University of Heidelberg approved the data collection and conduct of the present study (Sign 07/2013). As data for the database was entered anonymized, no additional patient consent was necessary (section 15, paragraph 1 of the code of medical ethics of the federal state of Baden-Württemberg). The prospectively maintained database for liver resections of the Department for General, Visceral, Transplantation and Pediatric Surgery in Heidelberg was searched for patients ≤ 20 years of age who were operated on between January 2009 and March 2020. Liver transplantations, as well as Kasai portoenterostomies, operations of choledochal cysts, endocystectomies for cystic echinococcosis, liver biopsies, and all other hepatobiliary operations without resection of liver parenchyma were excluded from the analysis. Since teenagers and young adults aged 16 to 20 share more clinicopathological characteristics with children, rather than adults undergoing hepatectomy, this age group was included into the analysis.

### Study Endpoints

To ensure a comprehensive and comparable investigation of patient outcome and care, the following endpoints were defined/applied: (1) Textbook Outcome (TO) and (2) Comprehensive Complication Index (CCI). By using composite measures, consisting of multiple individual factors, sufficient statistical power can be achieved in clinical studies with few patients (23). The new composite measure *Textbook Outcome* (21) aims to analyze surgical procedures more comprehensively for an improvement of patient care in different important aspects (20, 21). We defined TO in a pediatric surgery context for the first time. Criteria of our definition were based on those by Mehta et al., who first introduced TO for hepatobiliary surgery in adults (20). Accordingly, a Textbook Outcome requires:

1. No postoperative complication
2. No longer hospital stay (LOS) than the average number of days for the respective procedure (see details below)
3. No postoperative mortality
4. No readmission to any hospital within 90 days after discharge.

Only if all four conditions are fulfilled, a TO is achieved. In our study, the average length of hospital stay for the respective procedure was taken from the German Diagnosis-related Groups (G-DRG) inpatient reimbursement system. If a patient exceeded the average LOS by 1 day, it was classified as prolonged LOS and criteria for TO were not fulfilled.

As second endpoint, the Comprehensive Complication Index (CCI) was chosen. Developed and introduced by Slankamenac et al. in 2013, the CCI compiles all postoperative complications into one numeric value, ranging from 0 (no complication) to 100 (death of a patient) on a linear scale (22). The evaluation of the severity of a complication is based on the commonly applied Clavien-Dindo classification (24). In several studies, the CCI reached high sensitivity compared to established outcome measures and has been regularly applied in surgical studies I adults since its introduction (25, 26).

### Statistical Analyses

All statistical analyses were performed using R (version 3.6.2.) (27). Descriptive statistics were calculated: means with standard deviations (SD) or medians with ranges were given for continuous data and numbers with percentages for categorial data. Univariate associations were analyzed using chi-squared tests or linear regression. Multivariate logistic and linear regression models were used to identify independent predictors of TO and CCI. Effect sizes are expressed as odds ratio (OR) for binary outcome variables and as estimate *b* for continuous outcome variables. Factors included in the multivariate regression models were based on previous reports of pediatric liver resection populations and existing evidence on liver surgery in adult patients (references in the tables). As control variables, age, operation time and the extent of the liver resection were entered into the regression models. Given the wide range of circulating blood volume in a patient group comprised of infants and teenagers, total intraoperative blood volume loss is an inexact parameter. As proposed in our analyses, blood loss should always be measured as proportion of the estimated circulating blood volume in pediatric populations.

## Results

**Figure 1:**
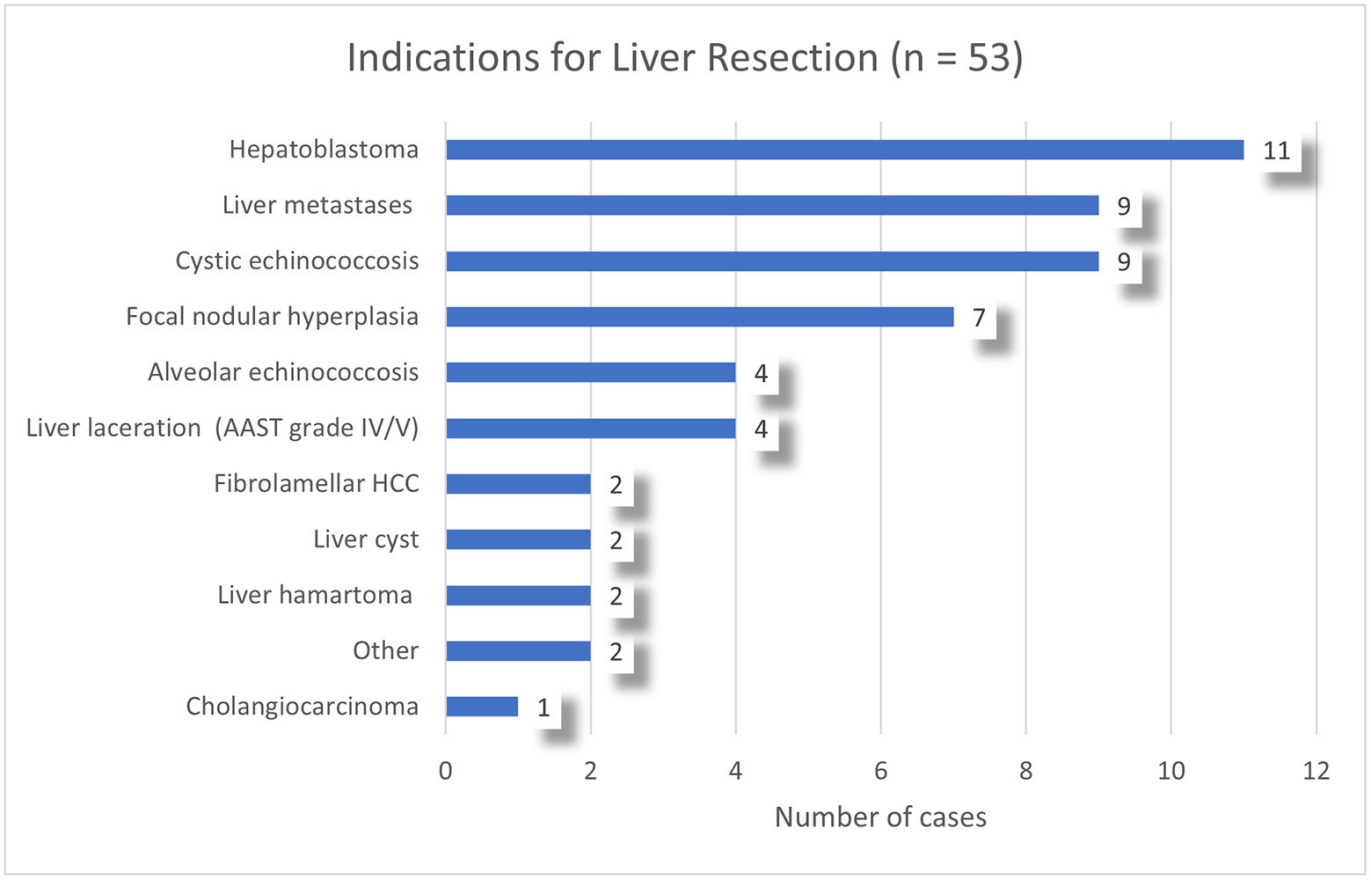
Indications for liver resections in the study population.

A total of 53 patients with a mean age of 11.3 years (SD 7.6) underwent a liver resection. 17 patients (32.1%) were underweight and three patients (5.7%) overweight (28). Congenital malformations or comorbidities were present in 15 patients (28.3%) (Table 1 and Figure 1).

**Table 1:** Patient characteristics.

	**Main population *n* = 53**	**Age ≤ 10 years *n* = 21**	**Age > 10 years *n* = 32**
Mean age [years] (SD)	11.3 (7.6)	2.9 (2.9)	16.8 (2.1)
Female gender	29 (55.0%)	8 (38.1%)	21 (65.6%)
Mean body weight [kg] (SD)	33.8	14.9 (14.2)	46.2 (12.8)
Mean BMI [kg/m^2^] (SD)	19.1 (4.2)	15.1 (2.8)	21.1 (3.3)
Underweight	17 (32.1%)	8 (38.1%)	9 (28.1%)
Premature birth	6 (11.3%)	6 (28.6%)	0
Congenital malformation	5 (9.4%)	5 (23.8%)	0
Patients with any comorbidity	15 (28.3%)	13 (61.9%)	3 (9.4%)
Hepatic	1 (1.9%)	1 (4.8%)	0
Renal	2 (3.8%)	2 (9.5%)	0
Cardiac	3 (5.7%)	3 (14.3%)	0
Pulmonary	2 (3.8%)	2 (9.5%)	0
Other	7 (13.2%)	4 (19.0%)	3 (9.4%)
**Symptoms before diagnosis**			
Abdominal pain	22 (41.5%)	4 (19.0%)	18 (56.2%)
None	18 (34.0%)	8 38.1%)	10 (31.2%)
Increase in abdominal diameter/painless abdominal mass	5 (9.4%)	5 (23.8%)	0
Weight loss	3 (5.7%)	1 (4.8%)	2 (6.3%)
Jaundice	3 (5.7%)	2 (9.5%)	1 (3.1%)
Others	2 (3.8%)	1 (4.8%)	1 (3.1%)
**ASA Score**			
ASA I	8 (15.1%)	2 (9.5%)	6 (18.8%)
ASA II	31 (58.5%)	11 (52.4%)	20 (62.5%)
ASA III	12 (22.6%)	7 (33.3%)	5 (15.6%)
ASA IV	1 (1.9%)	1 (4.8%)	0
ASA V	1 (1.9%)	0	1 (3.1%)
Primary liver tumor	28 (52.8%)	17 (81.0%)	11 (34.4%)
Malignancy	23 (43.4%)	12 (57.1%)	11 (34.4%)
Metastasized disease	10 (18.9%)	1 (4.8%)	9 (28.1%)
Previous abdominal surgery	20 (37.7%)	8 (38.1%)	12 (37.5%)
Preoperative tumor biopsy	15 (28.3%)	8 (38.1%)	7 (21.9%)
Neoadjuvant chemotherapy (before liver resection)	17 (32.1%, 60.8% of 23 with malignancy)	10 (47.6%, 83.3% of 12 with malignancy)	7 (21.9%, 64.6% of 11 with malignancy)
Adjuvant chemotherapy (after liver resection)	20 (37.7%, 87.0% of 23 with malignancy)	12 (57.1%, 100.0% of 12 with malignancy)	8 (25.0%, 72.7% of 11 with malignancy)
Mean tumor size [cm] (SD)	6.8 (3.2)	6.9 (2.7)	7.0 (3.9)
Tumor invasion major hepatic veins	11 (20.8%)	8 (38.1%)	3 (9.4%)
**Preoperative blood values**			
Preoperative elevated AFP	15 (28.3%)	12 (57.1%)	3 (9.4%)
Mean serum Bilirubin [mg/dl] (SD)	0.86 mg/dl	0.92 (1.85)	0.82 (0.79)
Mean hemoglobin [g/dl] (SD)	11.7 g/dl	11.1 (2.3)	12.1 (2.4)
Mean INR	1.04	1.05 (0.08)	1.04 (0.10)

### Operation Details

Open surgery was performed in 51 (96.2%) patients and laparoscopy was applied in two cases (3.8%). These surgeries were performed by interdisciplinary teams of expert hepatobiliary and pediatric surgeons, consisting of total of six different surgeons. Mean weight and age adjusted blood loss was 23.5% of circulating blood volume (SD 23.4). Severe intraoperative hemorrhage, defined as blood loss of more than 50% of the circulating blood volume (3), occurred in six patients. Three of those suffered from a traumatic liver laceration AAST Grade IV and V with difficult bleeding control. In another one of the six patients, a partial resection of the inferior vena cava due to tumor invasion (with patch reconstruction) led to substantial blood loss. Specific causes of the severe hemorrhage were not present in the two remaining patients, who underwent right trisectionectomy for POSTEXT III hepatoblastoma. Intermittent hepatic pedicle clamping (29) during the parenchyma transection phase was applied in all procedures. Stapler hepatectomy was the most applied resection technique with 30 cases (54.7%). The two laparoscopic resections were performed in two 14 and 17-years old girls. Both had tumors of the left lateral sector with suspected focal nodular hyperplasia, which was later confirmed by histopathology. They underwent left lateral sectorectomy with a mean operation time of 130 min and a mean blood loss of 10% of circulating blood volume. Both patients had a TO, and no recurrence or long-term sequelae occurred (Table 2).

**Table 2:** Details on surgical procedures.

	**Main population *n* = 53**	**Age ≤ 10 years *n* = 21**	**Age > 10 years *n* = 32**
Open surgery	51 (96.2%)	21 (100.0%)	30 (93.8%)
Median laparotomy	21 (39.6%)	8 (38.1%)	13 (40.6%)
Inverted L-incision	19 (35.8%)	6 (28.6%)	13 (40.6%)
Transverse subcostal incision	10 (18.9%)	6 (28.6%)	4 (12.5%)
Other incision type	1 (1.9%)	1 (4.8%)	0
Laparoscopic surgery	2 (3.8%)	0	2 (6.3%)
Mean operation time [min] (SD)	199.0 (85.2)	181.5 (82.9)	210.5 (84.8)
Mean blood loss [ml] (SD)	699.6 (945.7)	246.7 (358.8)	996.9 (1083.5)
Mean blood loss adjusted for circulating blood volume (SD)	23.5% (23.4)	24.5% (23.1)	22.8% (23.6)
Intraoperative transfusion of red cell concentrates [no. of cases]	17 (32.1%)	11 (52.4%)	6 (18.8%)
Mean amount of red cell transfusion [ml] (among those with transfusion) (SD)	670.6 (575.3)	212.7 (216.6)	1533.3 (906.8)
Intraoperative transfusion of fresh frozen plasma [no. of cases]	16 (30.2%)	10 (47.6%)	6 (18.8%)
Mean amount of fresh frozen plasma transfusion [ml] (among those with transfusion) (SD)	955.0 (828.1)	168.0 (216.5)	2266.7 (1236.5)
**Resection technique**			
Electronic monopolar knife/bipolar forceps	9 (17.0%)	4 (19.0%)	5 (15.6%)
Tissue sealing system (Ligasure™)	10 (18.7%)	6 (28.6%)	4 (12.5%)
Stapler hepatectomy	29 (54.7%)	7 (33.3%)	22 (68.8%)
Ultrasonic aspirator (CUSA™)	5 (9.4%)	4 (19.0%)	1 (3.1%)
**Resection type**			
Anatomical resection^*^	39 (73.6%)	16 (76.2%)	23 (71.9%)
Atypical resection^*^	21 (39.6%)	8 (38.1%)	13 (40.6%)
Right hemihepatectomy	10 (18.9%)	5 (23.8%)	5 (15.6%)
Right trisectionectomy	3 (5.7%)	2 (9.5%)	1 (3.1%)
Left hemihepatectomy	8 (15.1%)	2 (9.5%)	6 (18.8%)
Left trisectionectomy	1 (1.9%)	1 (4.8%)	0
Central liver resection	1 (1.9%)	1 (4.8%)	0
Left lateral sectionectomy	10 (18.9%)	2 (9.5%)	8 (25.0%)
Right posterior sectionectomy	1 (1.9%)	0	1 (3.1%)
Right anterior sectionectomy	1 (1.9%)	0	1 (3.1%)
Monosegmentectomy	4 (7.5%)	3 (14.3%)	1 (3.1%)
Atypical resection only	14 (26.4%)	5 (23.8%)	9 (28.1%)
*In-situ* split	1 (1.9%)	0	1 (3.1%)
Other visceral operation or vascular reconstruction	7 (13.2%)	1 (4.8%)	6 (18.8%)

* *Combined anatomical and atypical resection in one operation possible*.

Neither 30- nor 90-day mortality occurred. A major complication (≥ Clavien-Dindo grade III) occurred in 9 patients (17.0%). Twenty-eight patients (52.8%) developed at least one complication. Mean CCI was 17.2. Posthepatectomy liver failure (30) (PHLF, *n* = 9 of them: Grade A *n* = 5, Grade B *n* = 4, Grade C *n* = 0), posthepatectomy hemorrhage (31) (PHH, *n* = 9 of them: Grade A *n* = 6, Grade B *n*= 1, Grade C *n* = 2), posthepatectomy bile leakage (32) (PHL, *n* = 6 of them Grade A *n* =1, Grade B *n* = 1, Grade C *n* = 4) are presented in Table 3.

**Table 3:** Postoperative outcome.

	**Main population**	**Age ≤ 10 years *n* = 21**	**Age > 10 years *n* = 32**
30-day Mortality	0	0	0
90-day Mortality	0	0	0
**Textbook Outcome**	**23 (43.4%)**	**8 (38.1%)**	**15 (46.9%)**
Mean stay in intensive care unit [days] (SD)	4.5 (7.3)	3.2 (3.2)	5.4 (8.8)
Mean length of hospital say [days] (SD)	20.5 (24.0)	16.1 (10.1)	23.4 (29.6)
**Postoperative complications [no. of complications]**			
Clavien-Dindo grade I	7 (13.2%)	3 (14.3%)	4 (12.5%)
Clavien-Dindo grade II	26 (49.1%)	12 (57.1%)	14 (43.8%)
Clavien-Dindo grade III	9 (17.0%)	2 (9.5%)	7 (21.9%)
Clavien-Dindo grade IV	2 (3.8%)	0	2 (6.3%)
Clavien-Dindo grade V	0	0	0
**Mean CCI (SD)**	**17.2 (19.6)**	**15.6 (14.8)**	**18.2 (22.1)**
**Median CCI (range)**	**20.9 (0–83.8)**	**20.9 (0–44.9)**	**4.4 (0–83.8)**
Patients with at least one complication	28 (52.8%)	12 (57.1%)	16 (50.0%)
Major complications (≥ CD III)	9 (17.0%)	2 (9.5%)	7 (21.9%)
**Complication details**			
Posthepatecomty Bile Leakage			
Grade A	1 (1.9%)	0	1 (3.1%)
Grade B	1 (1.9%)	0	1 (3.1%)
Grade C	4 (7.5%)	0	4 (12.5%)
Posthepatectomy Liver Failure			
Grade A	5 (9.4%)	4 (19.0%)	1 (3.1%)
Grade B	4 (7.5%)	1 (4.8%)	3 (9.4%)
Grade C	0	0	0
Posthepatectomy Hemorrhage			
Grade A	6 (11.3%)	3 (14.3%)	3 (9.4%)
Grade B	1 (1.9%)	1 (4.8%)	0
Grade C	2 (3.8%)	0	2 (6.3%)
**All other complications**			
Wound healing disorder	3 (5.7%)	1 (4.8%)	2 (6.3%)
Superficial surgical site infection	2 (3.8%)	0	2 (6.3%)
Deep surgical site infection	4 (7.5%)	0	4 (12.5%)
Burst abdomen/ Incisional Hernia	4 (7.5%)	3 (14.3%)	1 (3.1%)
Ascites	2 (3.8%)	0	2 (6.3%)
Catheter infection	2 (3.8%)	2 (9.5%)	0
Urinary tract infection	3 (5.7%)	0	3 (9.4%)
Electrolyte disorder	2 (3.8%)	1 (4.8%)	1 (3.1%)
Pleural effusion	1 (1.9%)	0	1 (3.1%)
Pneumonia	1 (1.9%)	0	1 (3.1%)
Pneumothorax	1 (1.9%)	0	1 (3.1%)
Respiratory insufficiency	2 (3.8%)	1 (4.8%)	1 (3.1%)
Pancreatitis	3 (5.7%)	0	3 (9.4%)
Cholestasis /Cholangitis	1 (1.9%)	0	1 (3.1%)
Sepsis	1 (1.9%)	0	1 (3.1%)
Delirium	2 (3.8%)	0	2 (6.3%)
Nutritional insufficiency	3 (5.7%)	1 (4.8%)	2 (6.3%)
Thrombosis	3 (5.7%)	1 (4.8%)	2 (6.3%)
Other serious adverse events	2 (3.8%)	0	2 (6.3%)
**Resection margin (for tumor operations (malignant and benign) only** ***n*****=** **44)**			
R0	42 (95.5%)	14 (93.3%)	28 (96.6%)
R1	2 (4.5%)	1 (4.8%)	1 (3.1%)
R2	0	0	0

### Textbook Outcome

**Figure 2:**
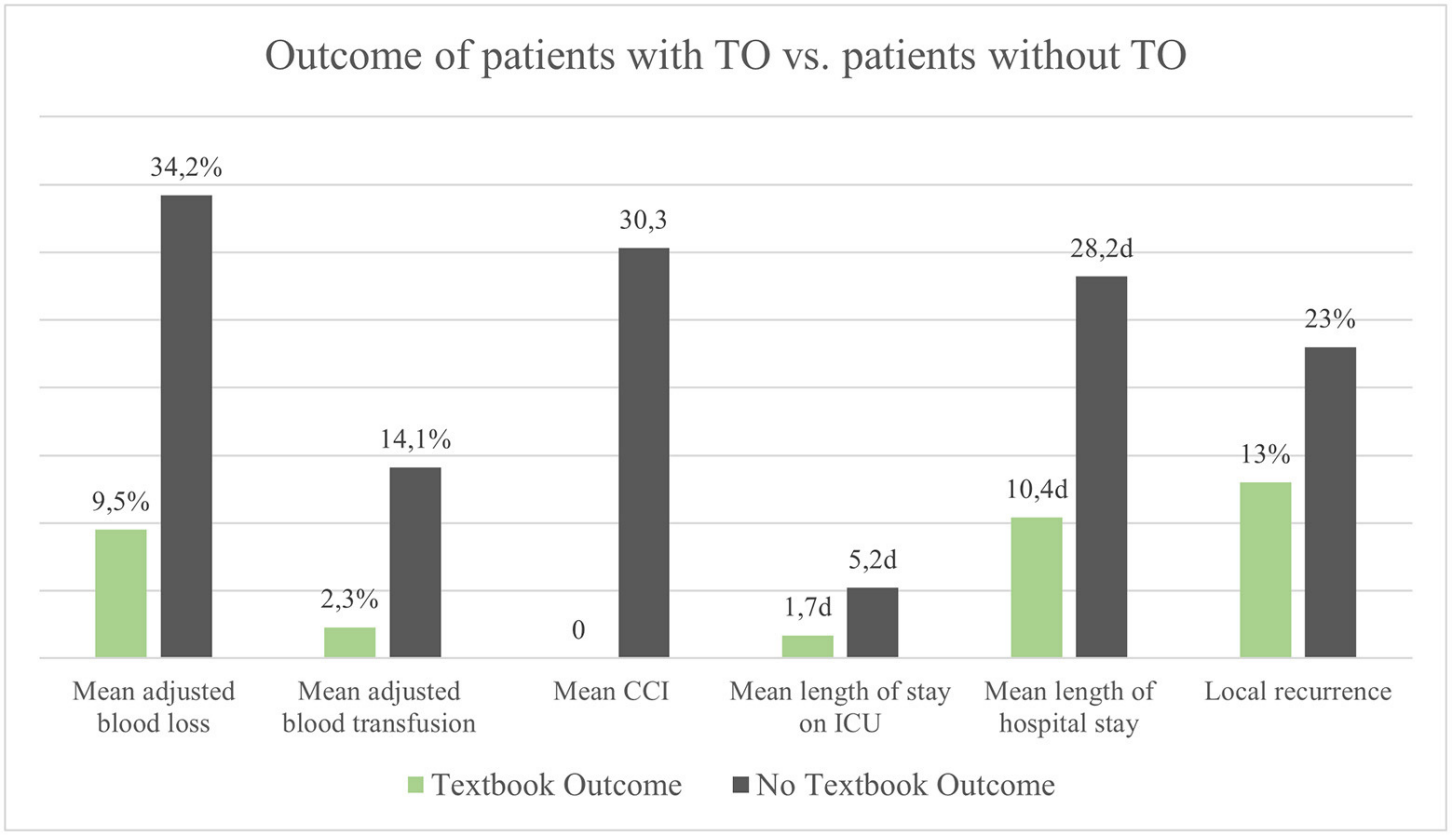
Comparison of outcomes of patients with TO vs. patients without TO; d, days.

Twenty-three patients had a TO (43.4%). Achievement of TO was significantly associated with lower values of CCI (*b* = −30.33, 95% CI [−37.44; −23.22], *p* < 0.001). On average, patients with TO had a shorter stay at the intensive care unit and a shorter length of hospital stay (Mean 1.7 vs. 5.2 days and 10.4 vs. 28.2 days, respectively). In univariate analyses, TO was significantly associated with the number of liver segments resected (OR 0.67, 95% CI [0.48; 0.91], *p* = 0.013), ASA score (OR 0.24, 95% CI [0.07; 0.63], *p* = 0.009), intraoperative blood loss (OR 0.99, 95% CI [0.99; 0.99], *p* = 0.021), intraoperative blood transfusion (OR 0.17, 95% CI [0.03; 0.63], *p* = 0.014), and previous abdominal surgery (OR 0.28, 95% CI [0.08; 0.90], *p* = 0.040). The rate of local recurrence was lower in patients with TO compared to those without TO (13 vs. 23%). There was no significant difference in univariate analyses between different resection techniques concerning the endpoints TO and CCI. Intraoperative blood loss adjusted for circulating blood volume proved to be an independent predictor of TO in a multivariable regression analysis controlled with 16 covariates (OR = 0.85, 95% CI [0.69; 0.97], *p* = 0.048) (Table 4 and Figure 2).

**Table 4:** Multivariable regression analysis for the endpoint Textbook Outcome.

**Factors**	**OR (95% CI)**	***p-*value**
**Patients analyzed:** ***n*** **=** **53**		
**Target factor: Textbook Outcome**		
Age	1.00 (0.99; 1.02)	0.508
Comorbidity	1.76 (0.11; 31.24)	0.682
Underweight	14.56 (0.87; 7.40^*^102)	0.108
ASA Score (33)	0.30 (0.02; 2.17)	0.275
Previous abdominal surgery	1.29 (0.07; 47.93)	0.875
Primary liver tumor (34)	4.22 (0.45; 64.61)	0.241
Malignancy (34)	0.93 (0.05; 25.83)	0.959
Neoadjuvant chemotherapy (35)	5.09 (0.02; 2.02^*^103)	0.557
Preoperative Hemoglobin level (36)	1.38 (0.87; 2.57)	0.219
Operation time	1.00 (0.99; 1.02)	0.614
Intraoperative blood loss^*^ **(****3**, **10**, **36****)**	**0.85 (0.69; 0.97)**	**0.048**
Severe intraoperative hemorrhage^†^ (3, 36)	7.15^*^10-5 (NA; 7.93^*^1082)	0.554
Intraoperative blood transfusion (37, 38)	0.95 (0.79; 1.11)	0.997
Anatomical resection performed (15)	0.73 (0.01; 39.61)	0.872
Number of segments resected	0.55 (0.13; 1.79)	0.342
Vascular or non-hepatic visceral resection performed (3)	0.32 (0.01; 21.09)	0.602

* *To adjust intraoperative blood loss and transfusion for age and bodyweight, the total amount in milliliters was divided by the estimated circulating blood volume (CBV) in milliliters. CBV was calculated using a method validated by Schmidt and Thews (39)*.

† *Severe hemorrhage was defined as intraoperative blood loss > than 50% of CBV (3)*.

### Comprehensive Complication Index

Mean CCI was 17.2 (SD 19.6, median 20.9, range 0–83.8). CCI was significantly associated with the length of hospital stay (*b* = 0.68, 95% CI [0.39; 0.97] *p* < 0.001). Intraoperative blood loss adjusted for circulating blood volume was an independent predictor of CCI (*b* = 0.77, 95% CI [0.22; 1.32], *p* = 0.008). In addition, low preoperative hemoglobin levels were significantly associated with higher CCI values (*b* = −3.42, 95% CI [−5.91; −0.93], *p* = 0.008) (Table 5).

**Table 5:** Multivariable regression analysis for the endpoint Comprehensive Complication Index.

**Factors**	**Estimate b (95% CI)**	***p*-value**
**Patients analyzed:** ***n*** **=** **53**		
**Target factor: Comprehensive Complication Index**		
Age	0.01 (−0.06; 0.09)	0.751
Comorbidity	7.44 (−7.60; 22.48)	0.322
Underweight	−6.86 (−17.52; 3.80)	0.200
ASA Score (33)	1.30 (−6.56; 9.15)	0.740
Previous abdominal surgery	0.89 (−13.56; 15.34)	0.901
Primary liver tumor (34)	−1.73 (−13.23; 9.78)	0.762
Malignancy (34)	−0.11 (−17.82; 17.60)	0.990
Neoadjuvant chemotherapy (35)	−6.38 (−32.76; 20.00)	0.627
**Preoperative Hemoglobin level** **(****36****)**	**−3.42 (−5.91;** **−0.93)**	**0.008**
Operation time	0.03 (-0.05; 0.11)	0.454
Intraoperative blood loss^*^ **(****3**, **10**, **36****)**	**0.77 (0.22; 1.32)**	**0.008**
Severe intraoperative hemorrhage^†^ (3, 36)	^−^14.10 (−43.47; 15.27)	0.337
Intraoperative blood transfusion (37, 38)	−0.41 (−1.01; 0.20)	0.180
Anatomical resection performed (15)	6.09 (−13.28; 25.47)	0.528
Number of segments resected	−0.61 (−6.20; 4.99)	0.827
Vascular or non-hepatic visceral resection performed (3)	0.04 (−20.94; 21.02)	0.997

* *To adjust intraoperative blood loss and transfusion for age and bodyweight, the total amount in milliliters was divided by the estimated circulating blood volume (CBV) in milliliters. CBV was calculated using a method validated by Schmidt and Thews (39)*.

† *Severe hemorrhage was defined as intraoperative blood loss > than 50% of CBV (3)*.

To compare our results with existing evidence, an overview of studies reporting surgical outcomes of pediatric liver resection is given in Table 6 (2–4, 10, 12, 13, 16–19, 40–44). Eleven single-center trials were found, with a median number of 27 reported cases in a median observation period of 15 year (1.8 cases per year on average). The studies included a total of 1,014 pediatric patients undergoing liver resection. The mean length of the recruitment period was 14 years. The reported complications rates ranged from 15.5 to 69.2%. Weighted mean postoperative morbidity and mortality across the 16 studies were 33.9% (SD 14.3) and 1.9% (SD 2.0), respectively (Table 6).

**Table 6:** Studies of pediatric patients undergoing liver resection with reported postoperative outcome.

**Year of publication**	**Author**	**Single- or multi-center study**	**Number of patients**	**Patient recruitment**	Postoperative morbidity^*^	**Surgery-related mortality**	**Percentage of major resections**
2002	Schnater et al.	Multi-center	128	1990–1994	23.0%	5.0%	64.8%
2006	Lin et al.	Single-center	14	1994–2002	35.7%	0.0%	100.0%
2007	Pham et al.	Single-center	45	1975–2005	29.0%	0.0%	69.0%
2007	Su et al.	Single-center	15	1988–2005	20.0%	0.0%	40.0%
2008	Needham et al.	Single-center	22	1994–2006	31.8%	0.0%	95.5%
2008	Szavay et al.	Single-center	67	1980–2000	Not reported	0.0%	88.0%
2009	Tannuri et al.	Single-center	47	1993–2008	15.5%	0.0%	93.6%
2010	Guérin et al.	Single-center	9	1996–2008	33.3%	0.0%	77.8%
2010	Malek et al.	Single-center	30	1990–2007	27.0%	3.3%	Not reported
2014	Zwintscher et al.	Multi-center	126	2009	30.7%	3.7%	Not reported
2015	Becker et al.	Multi-center	126	1999–2008	21.0%	Not reported	Not reported
2017	Busweiler et al.	Multi-center	73	1990–2013	57.5%	0.0%	85.2%
2017	Fuchs et al.	Single-center	27	1992 - 2015	22.2%	Not reported	100.0%
2018	Culbreath et al.	Multi-center	110	2012–2015	18.2%	0.0%	100.0%
2019	de Freitas et al.	Single-center	19	2010–2017	21.1%	5.3%	100.0%
2019	Liu et al.	Single-center	156	2006–2016	69.2%	1.9%	51.3%
Pooled Data			1014	Weighted mean:	33.9% (SD 14.3)	1.9% (SD 2.0)	

* *Most studies only reported 30-day and no 90-day morbidity and mortality*.

## Discussion

Liver resection in children is still associated with substantial morbidity and mortality (2–4, 10, 17). Interpretation of data is difficult due to the low level of evidence of the heterogenous, retrospective observational studies (15). Especially articles on risk factors and postoperative complications in pediatric liver resection are scarce. The aim of this study was to introduce and validate TO and the CCI as standardized measures of quality in pediatric liver resection. Objective and comparable reporting of surgical outcomes is essential for achieving improvements in pediatric surgery. For rare procedures such as liver resections in children, a joint effort of different centers is indispensable. If we manage to standardize reporting of outcome and thus make our results comparable, progress will be achieved.

No surgery-related mortality occurred in the presented population. Seventeen percent of the patients had a major complication (≥ Clavien-Dindo Grade III). In those studies that made information on the severity of postoperative complications available, similar or higher rates of major complications were reported (22 to 30%) (12, 13, 43). The overall morbidity rate of 52% is comparable to those reported by others (2, 4, 10, 17). Comparison to other studies is difficult here, too, as a standardized collection of postoperative events and classification of complications is missing in many studies. In studies that reported all grades of postoperative complications after pediatric liver resection, overall morbidity ranged from 46 to 69.2% (4, 10, 17).

43.4% of the patients had a TO, a composite measure first reported for a pediatric cohort in this study. Compared to results from adult populations, the outcome indicates a good quality of care (20). TO proved to be significantly associated with previously established risk factors in hepatobiliary surgery, such as ASA score, the number of liver segments resected, previous abdominal surgery, and intraoperative blood loss (33).

The CCI has been rarely used in pediatric surgical patients so far, despite its wide usage in adult surgical patients. Only two studies in pediatric populations, one on pediatric Nissen fundoplication and one on the outcome after repair of congenital duodenal obstruction, have applied the CCI previously (45, 46). The aim of the CCI is to give a comprehensive picture of postoperative complications and to reach higher sensitivity than methods assessing specific complications alone (22). In several studies, the CCI reached high sensitivity compared to established outcome measures and has been regularly applied in surgical studies on adults since its introduction (25, 26). The standardized numeric value allows for a quick and reliable comparison of different study populations.

In our study, patients with a TO had significantly a lower CCI, a finding that further strengthens the validity of the newly defined TO. Since TO comprises the length of hospital stay and demands rigorous criteria to be fulfilled, it is suited for objectively judging the quality of surgical procedures, especially concerning the short-term outcome. Moreover, by using composite measures with multiple individual factors, sufficient statistical power can be achieved in clinical studies analyzing few patients (23). This makes them ideal for studies on rare diseases or interventions, where study populations are inevitably smaller (23). However, full validation of TO and CCI for pediatric hepatobiliary surgery in a prospective setting is required, ideally in a future multi-center trial. So far, comparison of surgical quality has often been subjective and only clearly defined outcome measures such as TO allow for a comprehensive investigation and valid comparison of patient outcome and care (20, 21).

Intraoperative blood loss was significantly associated with both TO and CCI. In addition, low preoperative hemoglobin levels were significantly associated with higher CCI. It is of prime importance to measure blood loss adjusted for the circulating blood volume in pediatric surgery, as done in our study (39). Absolute values are of limited validity given the large differences regarding body weight in pediatric cohorts, that include infants and teenagers. In many studies, adjustment of blood loss is lacking, resulting in inadequate analysis of this possibly important risk or prognostic factor. Pediatric patients are particularly susceptible to blood loss and low circulating blood volume. Strong negative effects on the patients' outcome have been reported previously (10, 36, 37). Adverse effects of blood transfusions, such as lung injury, transfusion-related acute circulatory overload and hemolytic transfusion reactions, occur more often in pediatric patients than in the adult population, with associated mortality rates as high as 15–30% (36). On the other hand, compensating for loss of intravascular blood volume in pediatric surgical patients is vital, as it is the most common cause of circulatory arrest for children under general anesthesia (36). For tumor operations, intraoperative blood salvage techniques are contraindicated, making blood transfusion sometimes necessary in extended surgical procedures. A method to reduce blood loss that was used in our study cohort, was intermittent hepatic pedicle clamping. The application of the so-called Pringle's Maneuver (29) has been shown to be safe in pediatric liver resection (19). In contrast to many adult patients with liver tumors, pediatric patients undergoing liver resection do not usually have any preexisting liver disease or structural changes of liver parenchyma such as steatosis or cirrhosis (19), increasing the tolerance of temporary inflow reduction.

Within the last 15 years, only 15 single- and multi-center studies reported surgical outcomes, including at least 30-day morbidity and mortality after liver resection in a pediatric population. Many studies on pediatric liver surgery have not focused on postoperative complications, often resulting in insufficient postoperative outcome data. In the reviewed single-center studies, surgery-related mortality ranged from 0 to 1.9% and postoperative morbidity rates were between 15.5 and 69.2% (4, 10, 13, 41). Liu et al. identified extent of resection, application of Pringle maneuver and estimated total blood loss as independent predictors for surgery-related complications, with the most common being infections, bile leakage and ascites (10). In most of the registry or multi-center studies, no-surgery related risk factors were investigated, and outcome measures were heterogenous (2, 3, 14, 17, 40). Moreover, most single-center studies had observation periods of more than 10 years (median 15), and comparatively few cases investigated (1.8 pediatric liver resection per year on average). In the present study, 4.8 pediatric liver resections per year were performed. To increase the experience at specialized referral centers, the ongoing efforts to centralize these challenging cases should be further promoted.

This study has some limitations. First, the retrospective study design introduces selection bias. Thus, results and conclusions must be assessed and further validated in prospective trials. Second, while our single-center cohort of pediatric patients undergoing liver resection represents one of the largest hitherto reported, the absolute number is relatively small compared to liver surgery studies in adults. Nevertheless, this study presents a cohort of children undergoing liver resection with representative patient characteristics, and the statistical power was increased by applying the composite outcome measures as endpoints (23). Major strength of the study is the definition of TO and the first-ever application and validation of CCI in pediatric hepatobiliary surgery. The results show that these outcome measures are easily applicable, valid, and will allow for a better comparison of surgical quality in pediatric liver surgery.

## Conclusion

Evidence on comparable outcomes and risk factors in pediatric liver resection is low. The present study is the first to define and investigate the composite outcome measures Textbook Outcome (TO) and Comprehensive Complications Index (CCI) in a pediatric population. Both showed clear association with previously established risk factors in liver surgery and were significantly associated with each other. They proved to be suited outcome measures and will allow for a better comparison of results, higher transparency and thus provide the basis for improvement of patient care in pediatric liver surgery. Further validation of TO and CCI in prospective multi-center trials investigating outcomes in pediatric hepatobiliary surgery is needed.

Another conclusion from our study is that intraoperative blood loss is an important risk factor in pediatric liver resection and, most importantly, should generally be measured as proportion of the circulating blood volume. Given the wide range of the patient's body weight and thus circulating blood volume in pediatric cohorts, absolute blood loss has minor relevance and is not a valid risk factor in pediatric surgery.

To increase the base of evidence and professionalization in pediatric liver resection, nationwide and international registries should be established and centralization at specialized institutions be further promoted. The Children's Hepatic Tumors International Collaboration (CHIC) is one example for such projects (47). However, surgical aspects might be underrepresented in general oncological registries and need particular attention. For future studies, standardized and comparable outcome measures, as defined in our study, are an indispensable requirement on the way towards an improvement of surgical quality in pediatric liver surgery.

## Data Availability Statement

The raw data supporting the conclusions of this article will be made available by the authors, without undue reservation.

## Ethics Statement

The studies involving human participants were reviewed and approved by Ethikkommission der Medizinischen Fakultät Heidelberg, Alte Glockengießerei 11/1, 69115 Heidelberg, Germany. Written informed consent from the participants' legal guardian/next of kin was not required to participate in this study in accordance with the national legislation and the institutional requirements.

## Author Contributions

JF, KH, AM-L, MK, PG, GF, PP, and AM contributed to conception and design of the study. JF, AM-L, and KH extracted the data and performed the statistical analysis. JF and AM-L were responsible for the figures. JF wrote the initial draft of the manuscript. AM-L, KH, and PP wrote sections of the manuscript. All authors substantially contributed to manuscript revision, read, and approved the submitted version.

## Conflict of Interest

The authors declare that the research was conducted in the absence of any commercial or financial relationships that could be construed as a potential conflict of interest.

## Abbreviations

AFP: Alfa fetoprotein
ASA: American Society of Anesthesiologists
CCI: Comprehensive Complication Index
CI: Confidence interval
FNH: Focal nodular hyperplasia
HB: Hepatoblastoma
INR: International normalized ration
LOS: Length of hospital stay
OR: Odds ratio
PHLF: Posthepatectomy liver failure
SD: Standard deviation
TO: Textbook Outcome
WHO: World Health Organization.
